# Fermented blueberry juice extract and its specific fractions have an anti-adipogenic effect in 3 T3-L1 cells

**DOI:** 10.1186/s12906-016-1519-9

**Published:** 2017-01-06

**Authors:** Mayra L. Sánchez-Villavicencio, Melinda Vinqvist-Tymchuk, Wilhelmina Kalt, Chantal Matar, Francisco J. Alarcón Aguilar, Maria del Carmen Escobar Villanueva, Pierre S. Haddad

**Affiliations:** 1 Natural Health Products and Metabolic Diseases Laboratory and CIHR Team in Aboriginal Anti-diabetic Medicines, Department of Pharmacology and Physiology, Faculty of Medicine, Université de Montréal, C.P. 6128 Succ. Centre-ville / P.O. Box 6128, Downtown Postal Station, Montreal, H3C 3 J7 QC Canada; 2 Postgrade Program in Experimental Biology, Division of Health and Biological Sciences, Metropolitan Autonomous University of Iztapalapa, Mexico DF, Mexico; 3 Department of Health Sciences and Biological Sciences, Metropolitan Autonomous University of Iztapalapa Laboratory of Pharmacology, D.C.B.S, Mexico DF, Mexico; 4 Atlantic Food and Horticulture Research Center, Agriculture and Agrifood Canada, 32 Main St. Kentville, Nova Scotia, B4N 1 J5 Canada; 5 School of Nutrition Sciences, University of Ottawa, Ottawa, K1N 7 K4 ON Canada

**Keywords:** Obesity, Adipogenesis, Insulin signaling, Fermented blueberry extract

## Abstract

**Background:**

Obesity and Type 2 diabetes have reached epidemic status worldwide. Wild lowbush blueberry (*Vaccinium angustifolium* Aiton) is a plant of the North American Aboriginal traditional pharmacopeia with antidiabetic potential, especially when it is fermented with *Serratia vaccinii*.

**Methods:**

A phytochemical fractionation scheme was used to identify potential bioactive compounds as confirmed by HPLC retention times and UV–Vis spectra. 3 T3-L1 cells were differentiated for 7 days with either Normal Blueberry Extract (NBE), Fermented Blueberry Extract (FBE/F1), seven fractions and four pure compounds. Triglyceride content was measured. Examination of selected intracellular signalling components (p-Akt, p-AMPK) and transcriptional factors (SREBP-1c and PPARγ) was carried out by Western blot analysis.

**Results:**

The inhibitory effect of FBE/F1 on adipocyte triglyceride accumulation was attributed to total phenolic (F2) and chlorogenic acid enriched (F3-2) fractions that both inhibited by 75%. Pure compounds catechol (CAT) and chlorogenic acid (CA) also inhibited adipogenesis by 70%. Treatment with NBE, F1, F3-2, CAT and CA decreased p-AKT, whereas p-AMPK tended to increase with F1. The expression of SREBP1-c was not significantly modulated. In contrast, PPARγ decreased in all experimental groups that inhibited adipogenesis.

**Conclusions:**

These results demonstrate that fermented blueberry extract contains compounds with anti-adipogenic activity, which can serve to standardize nutraceutical preparations from fermented blueberry juice and to develop novel compounds with anti-obesity properties.

## Background

According to the WHO, global estimates of obesity rates in 2014 showed that more than 1.9 billion adults were overweight whereas 600 million were experiencing obesity [1]. Similar to many countries, Canada has experienced a substantial increase in the prevalence of obesity [2, 3]. Obesity and Type 2 diabetes have increased especially in Canadian Aboriginal populations. A report by the Organisation for Economic Co-operation and Development suggested that in some countries, including Canada, the prevalence of obesity will continue to rise at a predicted rate of 4–5% per year [2].

Adipocyte differentiation, or adipogenesis, implicates the accumulation of cellular lipid and is regulated by genetic and growth factors as well as hormones, notably by insulin [4]. Insulin is a major anabolic regulator of energy homeostasis and its signalling pathways implicate the serine/threonine-specific protein kinase Akt. Knockout models of the different Akt isoforms (Akt1 and Akt2) demonstrated their essential role in regulating adipogenesis [5] as well as glucose metabolism in the body [6]. Thus, Akt can drive adipogenesis. Its role includes the phosphorylation and regulation of a large number of substrates involved in several biological processes [7]. Adipocyte differentiation is also mediated by the temporally modulated expression of several transcription factors, notably PPARγ (peroxisome-proliferator-activated receptor gamma) that is a key regulator of this transcriptional program [8].

Another important enzyme involved in fat metabolism is the insulin-independent AMP-activated protein kinase (AMPK). It acts as a metabolic master switch regulating several intracellular systems, including the cellular uptake of glucose, the β-oxidation of fatty acids and mitochondrial biogenesis, which appear highly sensitive to energy status. Upon activation, AMPK increases cellular energy levels by inhibiting anabolic pathways (fatty acid synthesis, protein synthesis) and stimulating catabolic pathways (fatty acid oxidation, glucose transport) [9].

The use of natural health products as complementary or alternative approaches to existing medications is growing in popularity for the treatment and management of obesity and related diseases such as type 2 diabetes. Natural and synthetic agents can exert anti-obesity effects by increasing lipolysis in white adipocytes and by blocking adipocyte differentiation [10]. Notably, fermented blueberry extract was previously shown to inhibit triglyceride accumulation during adipogenesis of 3 T3-L1 cells [11], which could constitute a potential anti-obesity action. Such bio-transformed blueberry extract was also found to contain a much higher content in total phenolics and it was able to increase AMPK phosphorylation and glucose uptake in muscle cells and adipocytes [11, 12]. However, the compounds responsible for these effects still remain unclear, as does the participation of Akt in this process.

Consequently, the principal aim of this study was to identify potential bioactive components of fermented wild blueberry extract using 3 T3-L1 cells. For this purpose, we carried out phytochemical fractionation of fermented blueberry extract to generate semi-purified fractions and isolate active compounds that could be responsible for the observed inhibitory effect on adipogenesis. We also began addressing the potential molecular mechanisms that could underlie such an anti-adipogenic action by assessing the protein expression of selected key regulatory elements.

## Methods

### Chemicals and biochemicals

Dexamethasone (DXM), bovine pancreatic insulin, 3-isobutyl-1-methylxanthine (IBMX), dimethyl sulfoxide (DMSO) were purchased from Sigma-Aldrich. Rosiglitazone came from Alexis Biochemicals (Hornby, ON). Dulbecco’s Modified Eagle Medium was from Wisent Inc. (St-Bruno, QC). For the measurement of triglyceride content, the AdipoRed reagent was used (Lonza Walkersville Inc., Walkersville, MD). We also used the Western Lightning ECL from Perkin Elmer (Waltham, MA). Aminoimidazole carboxamide ribonucleotide (AICAR) was purchased from Toronto Research Chemicals (Toronto, ON). The measurement of protein was carried out with an assay kit from Bio-Rad (Mississauga, ON).

### Preparation of wild blueberry extract

Frozen wild blueberry fruit (*Vaccinium angustifolium* Aiton) was obtained from Oxford Frozen Foods Ltd (Oxford, NS). It represents a uniform blend of a large number of genotypes coming from several producers in Northeastern United States and Canada. In this respect, it accurately reflects the material that is normally used to prepare commercial wild blueberry juice. Notwithstanding, our laboratory analysis has confirmed that cultivar types do not affect the fermentation profile significantly.

The extract was prepared by blending such wild blueberry fruit (100 g) with an equivalent quantity (100 g) of Minimal Broth Davis without dextrose (MM) (Difco Laboratories, Detroit, MI). The preparation was centrifuged to remove insoluble particles. The resulting extract was sterilized using 0.22 μm Express Millipore filter (Millipore, Etobicoke, ON) and fermented with *Serratia vaccinii* bacteria as described [12]. Normal blueberry extract (NBE) was processed in an identical manner but was not fermented with the bacteria. Portions of NBE and the fermented blueberry extract (FBE) were freeze-dried and used for chemical analysis.

### Phytochemical fractionation of FBE

Fractions were prepared by a multi-step process (Table 1) that started with material that was either NBE or NBE after fermentation with *Serratia vaccinii* to produce FBE. As a first fractionation step, FBE (identified as F1; Table 1) was loaded in batches of 500 ml onto 29.5 × 5 cm chromatography columns (pre-conditioned with 1 column volume of methanol then 2 column volumes of water) containing Waters preparative C18 resin (125 Å, 55–105 μm). These were washed with 2 column volumes of water to remove sugars and organic acid (discarded). Phenolic compounds were eluted from the column using 1.2 column volumes of 100% ethanol containing 13 mM trifluoroacetic acid (Sigma Aldrich, ON). This ethanol eluent, which contained all the phenolics from the FBE starting material, was dried using rotary evaporation and lyophilisation. This total phenolic fraction from FBE was called F2.

**Table 1:** Fractionation of fermented blueberry extract (F1). The major component (s) in each fraction (F) are indicated

Fraction	Major component (s)	Starting material	Column resin	Eluant
F1 (FBE)	Sugars, organic acids, growth media, phenolics			
F2	phenolics	F1	C_18_	EtOH
F3-1	Gallic acid, catechol, protocatechuic acid	F2	C_18_	12% EtOH
F3-2	Chlorogenic acid	F2	C_18_	12% EtOH
F3-3	Flavonoids	F2	C_18_	80% EtOH
F4-1	Anthocyanins	F3-3	LH20	25% EtOH
F4-2	Heteropolymers	F3-3	LH20	50% EtOH
F4-3	Proanthocyanidins	F3-3	LH20	70% EtOH

For the second fractionation step of the FBE, one portion of F2 was dissolved in water and applied to another preconditioned C18 column to generate Fraction F3-1 that contained low MW phenolic compounds. This was done by selective elution using 4 column volumes of aqueous 2.06 M (12.5%) ethanol containing 0.16 M HCl (Ricca Chemical Company, Arlington, TX). F3-1 compounds recovered in this step were identified by HPLC by comparing retention times and UV–Vis profiles of the peaks to pure standards. The next fraction, called F3-2, was produced by passing through the same C18 column an additional 2 column volumes of aqueous 2.06 M (12.5%) ethanol containing 0.16 M HCl. This F3-2 fraction was rich in chlorogenic acid, which was confirmed by HPLC. The remaining bound materials were eluted using 0.16 M HCl and 13.7 M (80%) ethanol in water to produce fraction F3-3 (Table 1). The F3-1, F3-2 and F3-3 fractions were dried using rotary evaporation and then freeze dried.

To produce three additional and final fractions, one portion of F3-3 was dissolved in 4.28 M (25%) ethanol and applied to a 34.5 × 5 cm column of Sigma-Aldrich lipophilic Sephadex LH-20 (25–100 μm). The first fraction from Sephadex LH-20 was obtained by washing with 7 column volumes of 4.28 M (25%) ethanol. This fraction, called F4-1, was enriched in anthocyanins. The same Sephadex LH20 column was then washed with 3 column volumes of 8.56 M (50%) ethanol to yield a fraction enriched in phenolic heteropolymers and called F4-2. The last Sephadex LH20 fraction was eluted using 3 column volumes of 9.53 M (70%) acetone. This fraction was enriched in proanthocyanidins and called F4-3. The three fractions obtained from the Sephadex LH20 column (F4-1, F4-2 and F4-3) were dried using rotary evaporation and lyophilisation. All fractions were examined on HPLC and four pure compounds of interest were identified, namely catechol (CAT), protocatechuic acid (PA), gallic acid (GA) and chlorogenic acid (CA), which were subsequently purchased from Sigma-Aldrich (Oakville, ON).

### Cell culture

Murine 3 T3-L1 pre-adipocyte cells were obtained from ATCC (Manassas, VA) and used between passages 5 and 8. They were grown in Dulbecco’s modified Eagle’s medium (DMEM), containing 10% bovine calf serum until confluent at 100%. Two days after confluence (Day 0), the cells were stimulated to differentiate with DMEM containing 10% fetal bovine serum (FBS), 500 nM insulin, 1 μM Dexamethasone and 250 μM IBMX for 2 days (Day 2) (Short Term Medium). Cells were maintained in 10% FBS/DMEM with 500 nM insulin for another 4 days (Long Term Medium) at which time >90% of wells showed mature adipocytes with accumulated fat droplets. Cells were maintained at 37 °C in a humidified 5% CO_2_ atm and differentiated for a total of 7 days with media change every 2 days.

### Evaluation of cytotoxicity

Cell viability was assed through a Cytotoxicity Detection Kit that was purchased from Roche (South San Francisco, CA). 3 T3-L1 pre-adipocytes were seeded in 24-well plates and cultured to 100% confluence in culture medium and treated for 7 days as described above with various component concentrations (5, 10 and 15 μg/ml of NBE, FBE/F1, specific fractions and pure compounds). Cell culture media for each condition were collected separately (released LDH) and then cells were lysed with culture medium containing 1% Triton X-100, for 10 min (intracellular LDH). All samples were collected in Eppendorf tubes and centrifuged at 250xg at 4 °C for 10 min. Fluorescence was measured (Wallac Victor2, Perkin-Elmer, Waltham, MA) at an emission wavelength of 590 nm. Results were expressed as the ratio of released LDH to total LDH (intracellular plus released), normalized to values obtained from cells treated with the vehicle control (0.1% DMSO). The optimal concentration was determined for each tested component and used for bioassays.

### Adipogenesis assay

NBE, FBE (F1), specific fractions (5 μg/ml), four pure compounds at 5 μg/ml (except catechol at 3 μg/ml), and rosiglitazone (10 μM; positive control) were dissolved in DMSO and added to the cells as of day 0 of differentiation. The final concentration of DMSO was kept at 0.1% throughout the differentiation period. Adipogenesis was assessed in the well-characterized 3 T3-L1 cell model by measuring the accumulation of triglycerides upon differentiation after treatments as described previously [13, 14], using the AdipoRed reagent according to the manufacturer’s instructions. Briefly, after washing each well twice with phosphate-buffered saline (PBS: 8.1 mM Na_2_HPO_4_, 1.47 mM KH_2_PO_4_, 137 mM NaCl, and 2.68 mM KCl; pH 7.4), 2 ml of PBS 1X containing 60 μl of AdipoRed reagent were added to each well and incubated for 10 min at room temperature. Fluorescence was measured (Wallac Victor2, Perkin-Elmer, Waltham, MA) at 485 nm excitation and 572 nm emission wavelengths. Results are reported as a percentage of the value obtained for the vehicle control (0.1% DMSO).

### Western blot analysis

Cells were cultured, treated and differentiated as described above for the adipogenesis assay. Cultured cells were homogenized in lysis buffer (50 mmol/L HEPES, pH 7.4, 150 mmol/L NaCl 5 mmol/L EGTA, 2 mmol/L MgCl_2_, 1% Triton X-100, 1% sodium deoxycholate, 0.1% SDS) containing protease inhibitors (2 mmol/L PMSF and Complete Mini-EDTA-free protease inhibitor cocktail tablets; Roche, Laval, QC) and phosphatase inhibitors (0.5 mmol/L NaF, 2 mmol/L Na_3_VO_4,_ 1 mmol/L Na_4_P_2_O_7_). The protein concentration of lysates was assessed by the Bradford colorimetric assay and 40 μg of total protein were loaded onto a 12% acrylamide gel. Samples were electro-transferred to nitrocellulose membrane (Bio-Rad Laboratories, Hercules, CA). Membranes were incubated overnight with primary antibodies to Akt and *p*-Akt, (1:250, Cell Signalling Technology, Danvers, MA) as well as *p*-AMPK, AMPK, SREBP-1c and PPARγ (1:250, Santa Cruz Biotechnology Inc., Dallas, TX) and beta-actin (1:1000, Cell Signalling Technology Inc., Danvers, MA). Incubation with anti-rabbit HRP-conjugated secondary antibody (Cell Signalling Technology Inc., Danvers, MA) was then carried out at 1:20000 dilution in TBST (1X) (50 mmol/L Tris, pH 7.4, 150 mmol/L NaCl, 0.5% Tween 20) plus 5% non-fat dried milk for 1 h at room temperature. Membranes were then washed with TBST 3X for 5 min and the blots were revealed using the Western Lightning ECL enhanced chemiluminescence (Enhanced Chemiluminescence Substrate for 1000 cm2 of membrane). Densitometry analysis was performed with General Electric Image Quant LAS 4000 mini scanner and Image J software (GE Healthcare Bio Sciences, Baie d’Urfé, QC).

### Statistical analysis

Results are presented as the mean ± SEM of 3 independent experiments carried out in triplicate. Statistical analysis was performed with Prism GraphPad software (La Jolla, CA). The data were analyzed by one-way analysis of variance (ANOVA) followed by Dunnett’s and/or Tukey’s multiple comparison test for statistically significant differences between groups, as appropriate. Statistical significance was set at a level of *p* < 0.05.

## Results

### Phytochemical fractionation scheme and selection of pure compounds

**Fig. 1:**
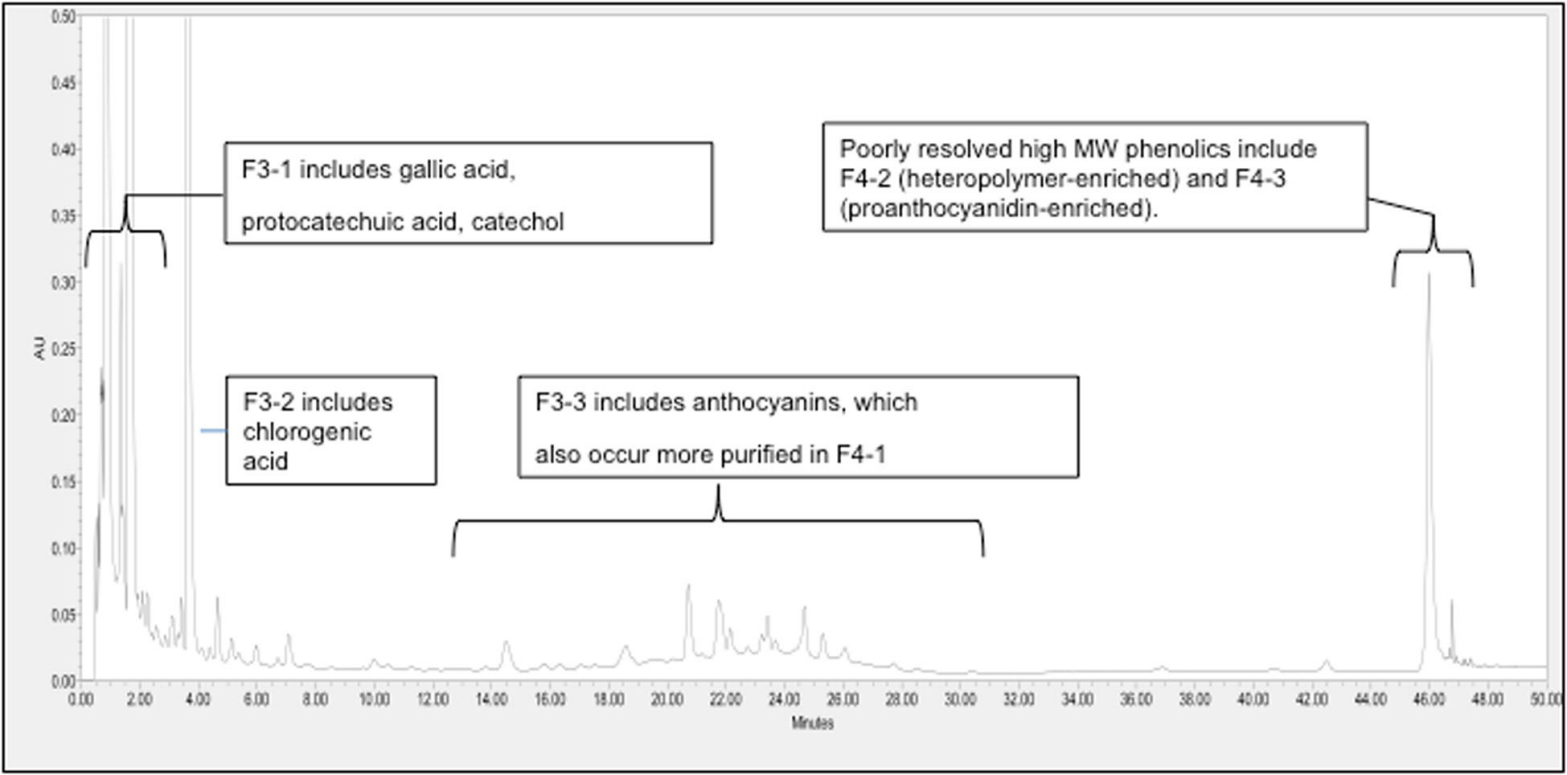
HPLC profile (280 nm) of fermented blueberry extract indicating the major components contained in fractions (see also Table 1)

Using a C18 column with varying ethanol and acid concentrations, sugars and organic acids were removed and several fractions were obtained as illustrated in Fig. 1 and listed in Table 1. This fractionation scheme was used previously to study the effect of NBE components on cardiomyocyte integrity [15] and was applied to FBE as a practical approach. Based on HPLC profiles (Fig. 1), fractions containing phenolic compounds (F2), whose sub-fractions contained gallic acid (GA), catechol (CAT) and protocatechuic acid (PA) (Fraction F3-1) as well as chlorogenic acid (CA) (Fraction 3-2), were of greatest interest [15].

### Cell viability

**Fig. 2:**
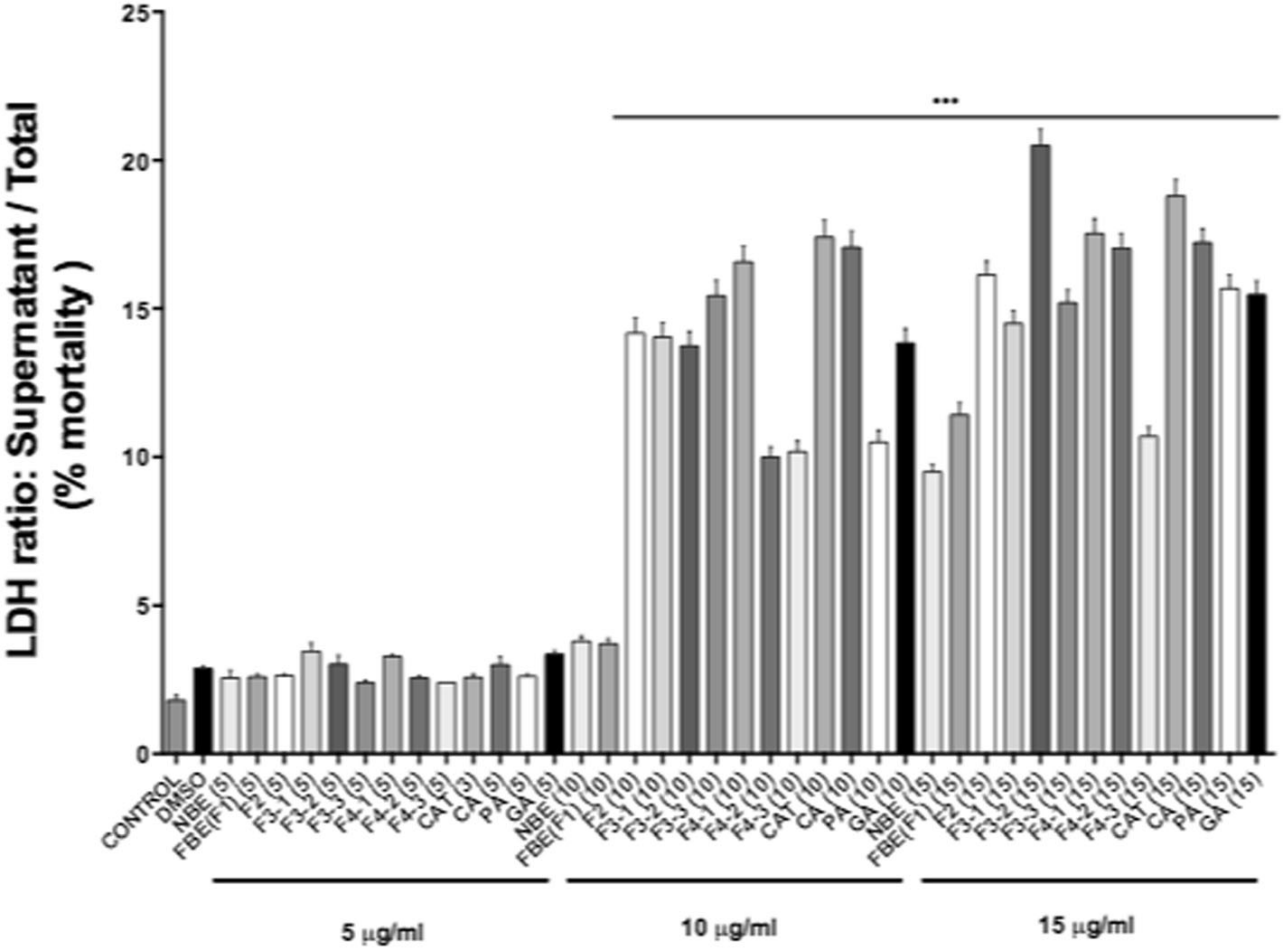
Lack of toxicity of optimal concentrations of fermented blueberry extract and components. 3 T3-L1 pre-adipocytes were seeded at a density of 2 × 10^4^ cells, cultured to 100% confluence and treated for 7 days with treatments as described above. Cytotoxicity was measured by LDH leakage. Medium LDH activity was expressed as a percentage of total enzyme activity (medium + lysate). The results are shown as the mean ± SEM. Significant differences compared to DMSO vehicle control were assessed by one way ANOVA; post hoc analysis with Dunnett’s multiple comparison test. *** Denotes statistically significant from vehicle control (*p* < 0.001)

Figure 2 presents the results of the LDH cytotoxicity assay. NBE and FBE as well FBE fractions and pure compounds were tested at 5, 10 and 15 μg/ml. As can be appreciated, NBE and FBE crude preparations induced LDH leakage that was similar to the DMSO control at concentrations of 5 and 10 μg/ml. In contrast, the optimal concentrations of other fractions and pure compounds were 5 μg/ml (except catechol at 3 μg/ml). In order to compare the various preparations at similar per weight contents, we also tested NBE and FBE at 5 μg/ml. Cells did not exhibit damage in response to such concentrations of test substances and were morphologically comparable to cells treated with the vehicle control 0.1% DMSO (data not illustrated). These concentrations were thus used subsequently for all in vitro assays.

### Adipogenesis assay

**Fig. 3:**
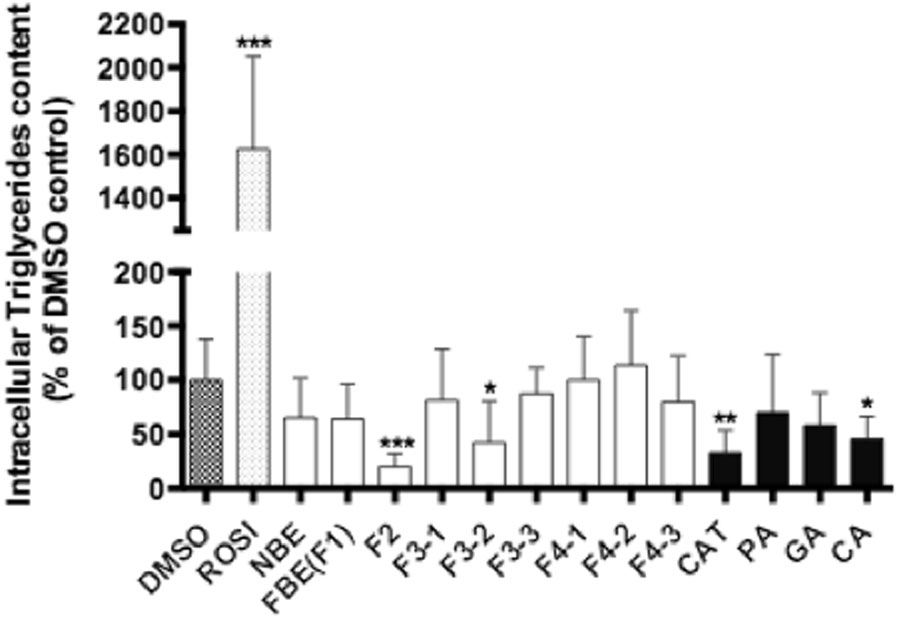
Fermented blueberry extract and fractions inhibit adipogenesis. 3 T3-L1 pre-adipocytes were seeded at a density of 2 × 10^4^ cells, cultured to 100% confluence and treated for 7 days with indicated treatments as described in Materials and Methods. Rosiglitazone was used as a positive control inducing differentiation (10 μM). DMSO 0.1% was used as vehicle control. The results are shown as the mean ± SEM. Significant differences compared to DMSO vehicle control were assessed by one-way ANOVA; post hoc analysis with Dunnett’s multiple comparison test. *Denotes statistically significant from vehicle control (*p* < 0.05). **Denotes statistically significant from vehicle control (*p* < 0.01). ***Denotes statistically significant from vehicle control (*p* < 0.001)

The amount of accumulated triglycerides was measured on day 7 in 3 T3-L1 cells treated with the specified concentrations of NBE, F1 and its specific fractions, as well as CAT, CA, PA and GA (7 fractions and 4 pure compounds). As illustrated in Fig. 3, only certain fractions of F1 were able to significantly inhibit TG accumulation. Notably, fractions F2 and F3-2 reduced triglyceride accumulation to 25 and 30% as compared to the vehicle control (set at 100%), respectively. Similarly, only two of the four pure compounds exerted a significant anti-adipogenic effect. Indeed, CA and CAT yielded adipogenic values that were 75 and 70% lower than DMSO.

### Western blot analysis

**Fig. 4:**
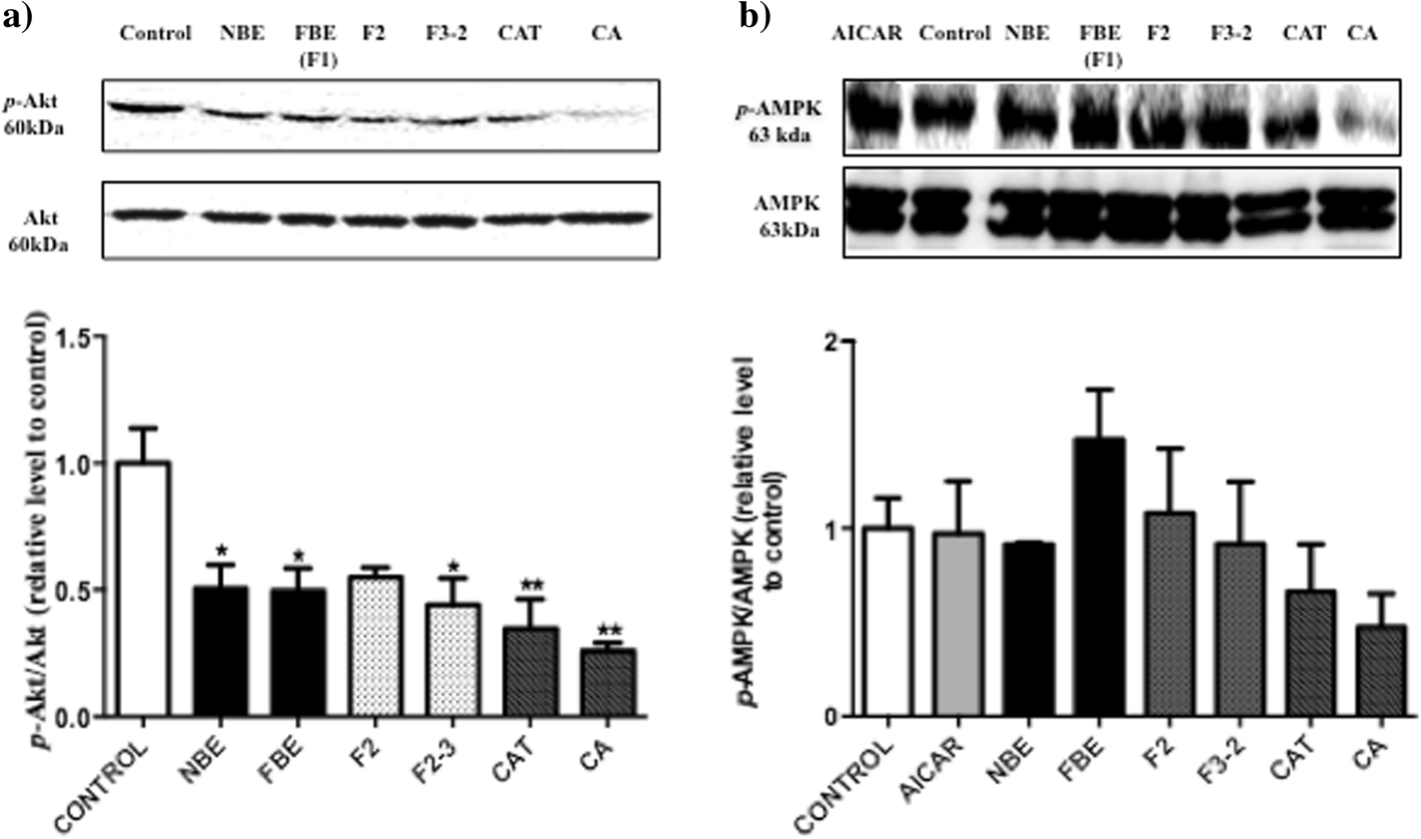
Effects of FBE and specific fractions on the phosphorylation of insulin-dependent Akt **a** and insulin-independent AMPK **b**. 3 T3-L1 preadipocytes were incubated with DMSO (0.1%) or 5 μg/ml of NBE, fractions F1, F2, F2-3 and two pure compounds CAT (3 μg/ml), CA (5 μg/ml) and induced to differentiate. The expression levels of key signalling proteins were measured after treatments by Western blot analysis; *p*-Akt, Akt **a** *p*-AMPK, AMPK **b**. Results are shown as the mean ± SEM. Significantly different compared to DMSO control *p < 0.05, **p < 0.01

Fractions and pure compounds with significant anti-adipogenic effect, namely F2, F3-2, CAT and CA, were analyzed by Western blot for cellular components involved in the control of adipogenesis and were compared with NBE and FBE (F1). As shown in Fig. 4a, all treatments that inhibited adipogenesis also decreased the phosphorylation of the insulin-dependent kinase Akt, with CA having the most prominent effect. In contrast, the phosphorylation of the insulin-independent kinase AMPK was not significantly affected by any of the treatments, albeit F1 had a tendency to increase it relative to vehicle control (Fig. 4b).

**Fig. 5:**
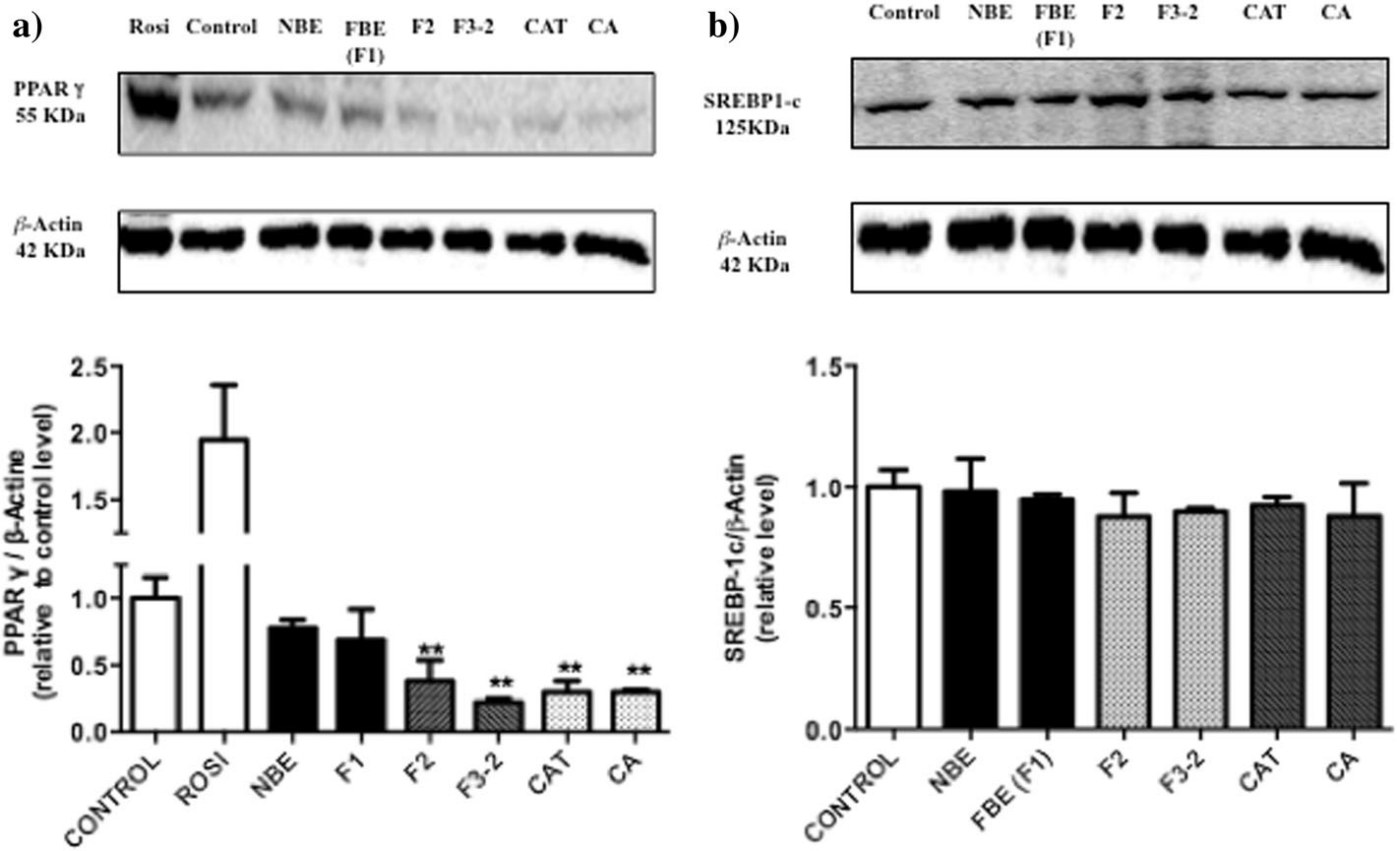
Effects of FBE and specific fractions on the expression of adipogenesis-related transcription factors. 3 T3-L1 preadipocytes were incubated with DMSO (0.1%) or 5 μg/ml of NBE, fractions F1, F2, F2-3 and pure compounds CAT (3 μg/ml), CA (5 μg/ml) and induced to differentiate. PPARγ **a** and SREBP-1c **b** expression levels were examined by Western blot. Protein expressions were normalized using β-actin as a reference. Results are shown as the mean ± SEM. Significantly different compared to DMSO control *p < 0.05, **p < 0.01

We next examined two selected key adipocyte-associated transcription factors. As illustrated in Fig. 5b, SREBP-1c was not significantly affected by any of the treatments. Conversely, all treatment conditions that significantly reduced adipogenesis, namely F2, F3-2, CAT and CA, markedly decreased the expression of PPARγ as compared to the vehicle control (Fig. 5a). As expected, the positive control Rosiglitazone significantly increased PPARγ expression (Fig. 5a).

## Discussion

Research in obesity is increasingly focusing on adipose tissue and adipogenesis. Indeed, adipose tissue appears to be involved with the development of the metabolic syndrome [16]. Food and medicinal plants are important sources of natural products that display great chemical diversity. This makes them excellent candidates for drug development, including for the treatment of obesity and other related diseases [17]. We previously observed that the fermentation of the juice from wild lowbush blueberries (*Vaccinium angustifolium* Aiton), with a bacteria (*Serratia vaccinii*) found on the fruit skin, was able to greatly increase the total phenolic content [12]. This biotransformation also conferred it potent anti-diabetic properties [11]. In the latter study, we also uncovered an inhibitory action of fermented blueberry juice on 3 T3-L1 adipogenesis, representing a putative anti-obesity action that was later confirmed in vivo [18]. In the present study, we sought to determine the biologically active compounds that could underlie such anti-adipogenic effects. We used an HPLC-based fractionation scheme that provided us with several fractions focused primarily on phenolic components. We notably identified four major compounds, namely gallic acid, catechol, protocatechuic acid and chlorogenic acid, which we also tested. We used the 3 T3-L1 cell line, one of the most reliable models for the study of adipogenesis [19].

As a first step, we freeze dried the fermented blueberry juice and the resulting extract, called F1, had a tendency to inhibit adipogenesis, albeit not to the extent that the intact fermented blueberry juice had done previously [11]. This may be explained by the different concentrations used in the two studies. In our previous study, intact fermented blueberry juice was administered to cells at a dose of total phenolics equivalent to 30 μM of gallic acid (GAE) [11]. In the present studies, we used FBE/F1 at the same weight-based concentration found to be optimal for the phytochemical fractions and pure compounds, in order to have a valid comparison. This concentration (5 μg/ml) amounts to 1.6 μM GAE, when the total phenolic content of FBE was taken into consideration. It is therefore almost 20 times less concentrated than that used in our previous study [11]. More surprisingly, when NBE was subjected to a similar freeze-drying step, it was found to also have a non-significant tendency to reduce adipogenesis. This was different than what was observed in our previous study, where NBE was without effect [11]. Further studies will be necessary to understand the potential effect of freeze-drying on the phytochemical composition of wild lowbush blueberry juice, notably in the context of adipogenesis. Indeed, other researchers, using cultivated highbush blueberry phenolics, have observed a concentration-dependent inhibition of adipogenesis in the 3 T3-F44A cell line [20].

Notwithstanding, we found that two sub-fractions of our fermented blueberry extract exhibited a significant inhibition of adipogenesis. The first, F2, was the fraction that contained all the phenolic compounds while the second, F3-2, was enriched in chlorogenic acid. This was confirmed by the corresponding activity of the pure compound (chlorogenic acid). Of the other pure compounds, only catechol was found to also inhibit adipogenesis significantly. This implies that chlorogenic acid and catechol may contribute to the anti-obesogenic potential of fermented blueberry juice.

We next began assessing some of the potential molecular mechanisms that can underlie the anti-adipogenic activity of fermented blueberry juice. Adipogenesis involves a complex and coordinated transcriptional cascade that leads to lipogenesis and TG accumulation [21, 22]. Insulin-dependent Akt signalling can trigger this cascade [5–7]. In contrast, insulin-independent AMPK signalling can inhibit adipogenesis [11]. In line with the inhibitory effect of fermented blueberry juice and its components, we found that F1 as well as sub-fractions F2 and F3-2 significantly inhibited the phosphorylation of Akt. This result is in line with the report by Song and collaborators who studied blueberry peel extracts and found an anti-adipogenic effect that was related to the inhibition of Akt phosphorylation [23].

On the other hand, F1 had a tendency to increase AMPK, although this effect failed to reach statistical significance. This implies that fermented blueberry products may diminish adipogenesis principally by reducing insulin-dependent signalling. Interestingly, our previous work had shown that fermented blueberry juice could stimulate AMPK in 3 T3-L1 adipocytes, an effect that was implicated in the stimulation of glucose transport observed in the same cells [11]. The implication of AMPK in the inhibition of adipogenesis by fermented blueberry juice may be less critical at the concentration used in the present studies.

The adipogenic process is a complex and highly regulated program of gene expression that involves several key transcription factors that include SREBP-1c and PPARγ [19, 24]. We therefore chose to determine the protein content of these two transcription factors as an initial step to assess potential underlying molecular mechanisms. Although treatment with sub-fractions F2 and F2-3 as well as pure compounds CAT and CA failed to significantly modulate SREBP-1c protein expression, they all had a tendency to reduce it. However, SREBP-1c is activated early in the transcriptional cascade such that our assessment near the end of adipogenesis may explain the low variations in protein expression that we observed. More detailed studies, including time-course and mRNA assessments will be necessary to verify the potential role of SREBP-1c in the effects of FBE on adipogenesis.

In contrast, the results of the current studies clearly show that the same active components of fermented blueberry juice induced a strong inhibition of PPARγ. Our results thus suggest that the negative modulation of PPARγ may be involved in the anti-adipogenic action of fermented blueberry juice. This is similar to what was observed by Song and collaborators, where blueberry peel extracts significantly reduced PPARγ [23]. Nevertheless, more detailed studies, notably addressing the modulation of the gene expression of several components controlling adipogenesis, will be necessary to confirm this point and determine if other factors than PPARγ are involved.

It is well known that a high phenolic content in the diet is associated with the prevention of certain chronic diseases, whereas phenolic acids have demonstrated antioxidant and anti-inflammatory properties [25]. Indeed, chlorogenic acid, a major component of green bean coffee extract, has been reported to reduce blood sugar levels and potentially exert an anti-diabetic effect [26]. It has also been implicated in weight loss in humans [27, 28] and recently shown to stimulate lipolysis in adipocytes in culture [29, 30].

The present studies suggest that chlorogenic acid may also exert its anti-obesity potential through the inhibition of adipocyte Akt phosphorylation and of the downstream transcription factor PPARγ, a major regulator of adipogenesis. On the other hand, catechol is a simple polyphenol found in a wide variety of plants. In the present studies, it was also found to significantly diminish adipogenesis through mechanisms involving an inhibition of Akt phosphorylation and a reduction in PPARγ. To our knowledge, this is the first instance where such biological activity has been reported for catechol.

## Conclusions

We identified chlorogenic acid and catechol as active components of fermented blueberry juice. These compounds can serve to further develop the potential of this fermented juice as a novel nutraceutical. Both compounds can also serve as templates for the development of novel alternative therapeutic agents against obesity and related diseases.

## Abbreviations

AICAR: Aminoimidazole carboxamide ribonucleotide
AKT: Serine/threonine-specific protein kinase
AMP: Adenosine monophosphate
AMPK: AMP-activated protein kinase
CA: Chlorogenic acid
CAT: Catechol
cm: Centimeter
DMSO: Dimethyl sulfoxide
DXM: Dexamethasone
FBE: Fermented blueberry extract
HPLC: High performance liquid chromatography
IBMX: 3-isobutyl-1methylxanthine
L: Liter
ml: Milliliter
NBE: Normal blueberry extract
PPAR: Peroxisome-proliferator–activated receptor
SREBPs: Sterol regulatory element-binding proteins
T2DM: Type 2 diabetes mellitus
*μ* g: Microgram
*μ* l: Microliter
